# Genotype Distribution of the *ACTN3* p.R577X Polymorphism in Elite Badminton Players: A Preliminary Study

**DOI:** 10.3390/genes14010050

**Published:** 2022-12-23

**Authors:** Javier Abián-Vicén, Pablo Abián, Alfredo Bravo-Sánchez, Inés Piñas-Bonilla, Beatriz Lara, Juan Del Coso

**Affiliations:** 1 Performance and Sport Rehabilitation Laboratory (DEPORSALUD), Faculty of Sport Sciences, University of Castilla-La Mancha, 45071 Toledo, Spain; 2 Faculty of Humanities and Social Sciences, Comillas Pontifical University, 28015 Madrid, Spain; 3 Faculty of Health Sciences, Universidad Francisco de Vitoria, 28223 Pozuelo de Alarcón, Spain; 4 Exercise Physiology Laboratory, Camilo José Cela University, 28692 Villanueva de la Cañada, Spain; 5 Centre for Sport Studies, Rey Juan Carlos University, 28943 Fuenlabrada, Spain

**Keywords:** elite athlete status, racquet sports, α-actinin-3, genetics, athletic performance, professional athlete

## Abstract

α-Actinin-3 is a protein with a structural role at the sarcomeric Z-line in skeletal muscle. As it is only present in fast-type muscle fibers, α-actinin-3 is considered a key mechanical component to produce high-intensity muscle contractions and to withstand external tension applied to the skeletal muscle. α-Actinin-3 is encoded by the gene *ACTN3*, which has a single-nucleotide polymorphism (p.R577X; rs1815739) that affects the expression of α-actinin-3 due to the presence of a stop codon. Individuals homozygous for the 577R allele (i.e., RR genotype) and RX heterozygotes express functional α-actinin-3, while those homozygous for the 577X (i.e., XX genotype) express a non-functional protein. There is ample evidence to support the associations between the *ACTN3* genotype and athletic performance, with higher frequencies of the 577R allele in elite and professional sprint and power athletes than in control populations. This suggests a beneficial influence of possessing functional α-actinin-3 to become an elite athlete in power-based disciplines. However, no previous investigation has determined the frequency of the *ACTN3* genotypes in elite badminton players, despite this sport being characterized by high-intensity actions of intermittent nature such as changes of direction, accelerations, jumps and smashes. The purpose of this study was to analyze *ACTN3* R577X genotype frequencies in professional badminton players to establish whether this polymorphism is associated with elite athlete status. A total of 53 European Caucasian professional badminton players competing in the 2018 European Badminton Championships volunteered to participate in the study. Thirty-one were men (26.2 ± 4.4 years) and twenty-two were women (23.4 ± 4.5 years). Chi-squared tests were used to analyze the differences in the distribution of *ACTN3* genotypes (RR, RX and XX) between categories and sexes. The *ACTN3* RR genotype was the most frequent in the sample of professional badminton players (RR = 49.1%, RX = 22.6% and XX = 28.3%). None of the badminton players ranked in the world’s top ten possessed the XX genotype (RX = 60%, RR = 40%). The distribution of the *ACTN3* genotypes was similar between male and female professional badminton players (men: RR = 45.2%, RX = 25.8% and XX = 29.0%; women: RR = 54.5%, RX = 18.2% and XX = 27.3%; χ^2^ = 0.58; *p* = 0.750). The distribution of the *ACTN3* genotypes in badminton players was different from the 1000 genome database for the European population (χ^2^ = 15.5; *p* < 0.001), with an overrepresentation of the RR genotype (*p* < 0.05) and an underrepresentation of the RX genotype (*p* < 0.01). In conclusion, the expression of functional α-actinin-3, associated with RR and RX genotypes in the *ACTN3* gene may confer an advantage for reaching the status of elite athlete in badminton, and especially the world’s top-ten ranking. Large-scale studies with different ethnic backgrounds are needed to confirm the association of the R allele of *ACTN3* with badminton performance.

## 1. Introduction

*ACTN3* is the gene that encodes for α-actinin-3, a sarcomeric protein expressed in type II (i.e., fast-type) muscle fibers [1]. The *ACTN3* gene has a common polymorphism, the p.R577X (c.1858C > T; rs1815739), which has a prevalence of more than 70% in the Americas and 44% in Europe [2]. This polymorphism is key to expressing functional α-actinin-3 as it implies a nonsense variant that produces the replacement of an arginine (R) with a premature stop codon (X) at amino acid 577, leading to an early end of the protein-coding sequence. As a result, individuals who are homozygous for the null X allele (i.e., XX genotype) are unable to express functional α-actinin-3 in fast-twitching muscle fibers [3]. In contrast, individuals homozygous for the R allele (i.e., RR genotype) or those with the RX genotype produce fully functional α-actinin-3. Interestingly, a dose–response relationship has been found in terms of protein expression, with RR individuals expressing more protein than RX individuals [4]. In *ACTN3* XX individuals, the deficiency of functional α-actinin-3 does not entail any medical condition, as the lack of α-actinin-3 in type II muscle fibers is compensated by a higher expression of α-actinin-2, an α-actinin isoform expressed in all types of muscle fiber [3]. However, the possession of the XX genotype has been linked with several consequences for exercise and sports performance, such as the underrepresentation of this genotype in elite athletes of sports with specialties of strength and power [5,6,7], a lower capacity of the skeletal muscle to resist strain [8,9], higher probability of exercise-related muscle-type injury [10,11] and a potentially higher incidence of ligament injury [12]. On the other hand, it has been suggested that the XX genotype might be beneficial for endurance sports based on animal studies carried out with *Actn3* knockout mice [13]. However, several studies have reported no increased frequency of the XX genotype in endurance athletes [14,15], with some exceptions [16], suggesting that the benefits of the XX genotype in animals may not be translated to humans.

The negative outcomes of possessing the XX genotype for power-based sports have been well established in recent reviews [17,18,19]. However, the role of the *ACTN3* R577X genotype in sports with an intermittent nature is not well understood. In intermittent sports, there is a contribution of both anaerobic and aerobic metabolism, although the role of each metabolism varies depending on several factors, such as the dimensions of the field/court where it is played, the duration of each rally/point and the recovery time between rallies/points, the duration of the matches and the possibility of making substitutions. For example, the frequency of XX tennis players was similar among professional and non-professional tennis players [20]. Likewise, the frequency of XX volleyball players and XX basketball players is similar in a sample categorized as elite athletes to a control population [21]. However, a meta-analysis of current studies about the influence of the R577X polymorphism on the athlete status in football concluded that the presence of XX elite players is lower than in control non-athlete populations [22]. These outcomes suggest that the influence of being α-actinin-3-deficient due to possessing the XX genotype in the *ACTN3* gene may be dependent on the characteristics of the sports, especially for those with an intermittent nature [23]. 

Badminton is an intermittent sport that combines high-intensity and explosive efforts (~11 s) with intervals of rest between points (~25 s), lasting approximately 45 min (average duration of a badminton match) [24]. During the rallies, badminton players must cover an area of 5.18 m × 6.70 m, hitting the shuttle before it touches the ground. On average, players perform 7–10 short and explosive movements per rally with an eccentric component to recover the position in the center of the court after hitting the shuttlecock [25]. The most important phases of badminton footwork, such as quick changes of direction, short accelerations, decelerations and lunges, are based on muscle power. Hence, most movement structures of badminton are associated with powerful contractions of the lower body and the upper body and are likely metabolically dependent on anaerobic pathways, as suggested by the high values of exercise-induced muscle damage after badminton matches [26,27]. With this background, badminton can be categorized as an intermittent sport with the contribution of anaerobic (during the game) and aerobic (during recovery) metabolism, but most movements associated with high performance in badminton are short and based on the production of high levels of power. To the authors’ knowledge, no previous study has determined the frequency of XX badminton players in a sample of elite athletes. Therefore, the purpose of this study was to analyze the *ACTN3* R577X genotype frequencies in professional badminton players to establish whether this polymorphism is related to badminton performance. Considering the nature of the physical efforts in badminton, we consider that the *ACTN3* R allele and the RR genotype will be overrepresented in professional badminton players, with a progressively lower presence of XX players in top tiers of the Badminton World Federation.

## 2. Materials and Methods

### 2.1. Participants

A total of 53 European Caucasian professional badminton players volunteered to participate in the study. From this sample, 31 participants were men (age = 26.2 ± 4.4 years; body mass = 73.6 ± 6.8 kg and height = 179.2 ± 6.0 cm) and 22 were women (age = 23.4 ± 4.5 years; body mass = 60.7 ± 5.9 kg and height = 166.5 ± 4.7 cm). Participants were informed and recruited in advance through email because they were classified to compete in the singles draw of the 2018 edition of the European Badminton Championship. The following inclusion criteria were established for participants: (a) professional badminton players pertaining to a first-division club in their respective national leagues; (b) performed regular badminton training of >2 h per day, >5 days per week for the prior six months; and (c) participation in the 2018 international circuit of the Badminton World Federation (BWF). The only exclusion criterion set was (a) having suffered a severe injury (>30 days of return to play) in the prior six months. The sample was categorized as world-class elite using the classification proposed by Swann et al. [28], and participants trained on average 22.4 ± 2.6 h/week, competed in international events and were ranked between positions 150 and 1 in the BWF ranking. In the study sample, there was 1 World Champion, 3 European Champions and 16 contestants in any edition of the Olympic Games. An a priori sample size calculation indicated that at least 39 participants were required to obtain a statistically significant difference in the frequency distribution of the *ACTN3* genotypes between two cohorts, for an effect size in w of 0.5 units, with a statistical power of 0.80 and a two-tailed α level of 0.05. The sample size was calculated using G*Power software (v.3.1.9.7, Germany), selecting goodness-of-fit as the statistical test. In the 2018 European Badminton Championship, there were a total of 48 players for the men’s singles tournament and 47 players for the women’s singles tournament; thus, the current study recruited 65.5% of men participants and 46.8% of women participants in the Championship. Before the onset of this investigation, all participants were informed about the purpose and procedures, and written informed consent was obtained from them. The procedures were approved by the Camilo José Cela University institutional ethics review committee in accordance with the code of ethics of the World Medical Association (Declaration of Helsinki). Players’ rights and confidentiality were protected during the investigation, and the genetic information was used only for the purposes of this research.

### 2.2. Procedures

Data were collected during the 27th edition of the European Badminton Championship. This tournament took place in Huelva (Spain) between 24 and 29 April 2018. All measurements were conducted by the same researchers. After enrollment, participants completed a questionnaire about personal and training information. Subsequently, anthropometric measurements (body mass and height; ±0.1 kg and ±0.01 m scale with stadiometer; Seca 711, Hamburg, Germany) were registered. Genomic DNA was obtained from cotton swab buccal according to a previously described protocol [29], and genotyping was performed in a certified genetics laboratory. To avoid contamination, recommendations for molecular genetics laboratories were followed, including physically isolated work area laboratories for each process (sample manipulation and extraction). After collection, the samples were refrigerated at 4 °C and shipped to the laboratory. Upon arrival at the laboratory, the extraction of genomic DNA was carried out by automatic extraction in QIACube equipment (QIAGEN, Venlo, The Netherlands) to obtain a solution with a DNA concentration of at least 25 ng/mL. This solution was frozen at −20 °C until genotyped, which was carried out within one week of laboratory arrival. During the genotyping process, the p.R577X polymorphism (rs1815739; c.1858C > T) in the *ACTN3* gene was genotyped using single-nucleotide primer extension (SNPE). For this process, the SNaPshot Multiplex Kit (Thermo Fisher Scientific, Waltham, MA, USA) was used with capillary electrophoresis fragment analysis in ABI3500 equipment (Applied Biosystems, Foster City, CA, USA). Genotyping was conducted using a TaqMan SNP Genotyping Assay (Applied Biosystems, Foster City, CA, USA [30]), and the reaction was performed in an Applied Biosystems 7500 Fast Real-Time PCR System (Applied Biosystems, Foster City, CA, USA). All analyses that did not offer a clear genotype assignment were repeated. In addition, reference samples (internal controls, blank samples and negative controls) and contamination monitoring in all steps were included. Positive controls for all genotypes were obtained from the Mexican branch of the CANDELA Consortium. The results were analyzed using 7500 Software v 2.0.5 (Applied Biosystems, Foster City, CA, USA). The genotyping success for this sample was 100%.

### 2.3. Statistical Analysis

The statistical analysis was performed with IBM SPSS Statistics 23.0 (SPSS, Chicago, IL, USA). Descriptive data are presented as frequencies and percentages for categorical variables and as mean ± standard deviation for continuous variables. A chi-square (χ^2^) test was used to verify that the genotype frequencies were in Hardy–Weinberg equilibrium (HWE). A χ^2^ test was also used to verify if the genotype frequency in our cohort of badminton players was different from the 1000 genome database of ethnically matched controls [31]. A sub-analysis was performed by dividing the sample of professional badminton players according to their best World Badminton Federation (BWF) ranking (i.e., Top 10, Top 20, Top 50 and Top 100). The differences in the distribution of the different genotypes of the R577X polymorphism in the *ACTN3* gene in the different tiers created by using players’ rankings were identified using χ^2^ tests, including adjusted standardized residual or Fisher’s exact test was when n < 40 (e.g., comparison between categories with Top 10, Top 20, Top 50 rankings and between-sex comparison). We also examined the magnitude of this distribution by a one-to-one comparison between the ranking position using Cramer’s V coefficients. A probability level of *p* < 0.050 was defined as statistically significant.

## 3. Results

Table 1 shows the frequency distribution of the different genotypes in the *ACTN3* R577X polymorphism in professional badminton players. The distribution of the *ACTN3* genotypes did not follow HWE (*p* < 0.001). Overall, the distribution of RR, RX and XX genotypes was similar in men and women groups (*p* = 0.769). However, the genotypic frequency in our sample of badminton players was different from the 1000 genome database (χ^2^ = 15.5; *p* < 0.001), with an overrepresentation of the RR genotype (expected = 32.6%; *p* < 0.05) and an underrepresentation of the RX genotype (expected = 48.3%; *p* < 0.01) in the professional badminton players.

**Figure 1:**
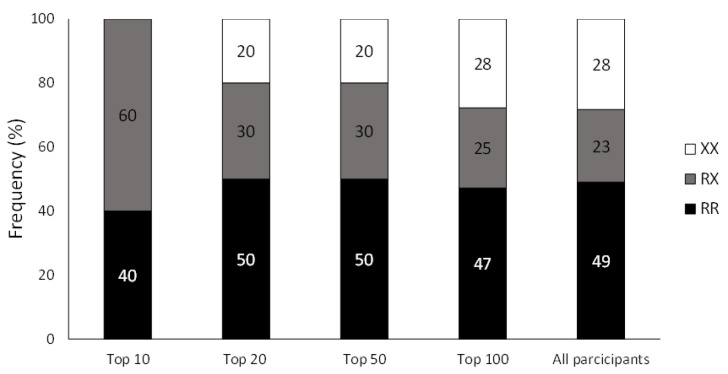
Frequencies of *ACTN3* genotypes in professional badminton players.

The distribution of genotypes according to the players’ World Badminton Federation (BWF) ranking is depicted in Figure 1. The frequency distribution of the *ACTN3* R577X polymorphism was similar (χ^2^ = 4.57; *p* = 0.802) among the different groups of ranking. No differences were found in the pairwise comparison (Table 2) between categories in the distribution of *ACTN3* genotypes. However, no XX player was present in the group of top ten players.

## 4. Discussion

The purpose of this investigation was to determine the distribution of the different *ACTN3* R577X genotypes in professional badminton players to establish whether this gene might be related to badminton performance. The main findings of this study were: (i) the *ACTN3* RR genotype was the most frequent in the sample of professional European badminton players; (ii) the genotypic frequency in professional badminton players was different with respect to the European population with an over-representation of the RR genotype and an under-representation of the RX genotype; (iii) none of the world top-ten badminton players possessed the *ACTN3* XX genotype; and (iv) the distribution of the *ACTN3* genotypes was similar between male and female professional badminton players. Collectively, the outcomes suggest that the expression of functional α-actinin-3, associated with the RR and RX genotypes, increases the likelihood of becoming elite in badminton players, especially to reach the top-ten ranking. The RR genotype may offer some benefits for badminton performance as its frequency in the sample of elite players was higher than in the non-athletic European population. On the other hand, the XX genotype may limit the possibility of reaching world’s top positions, likely due to the negative exercise phenotypes associated with this genetic variant [5,6,7]. In any case, these results are exploratory, and they should be confirmed by further investigation where performance phenotypes (such as muscle power, running speed, jump capacity, etc.) are compared in elite badminton players with different *ACTN3* genotypes.

**Figure 2:**
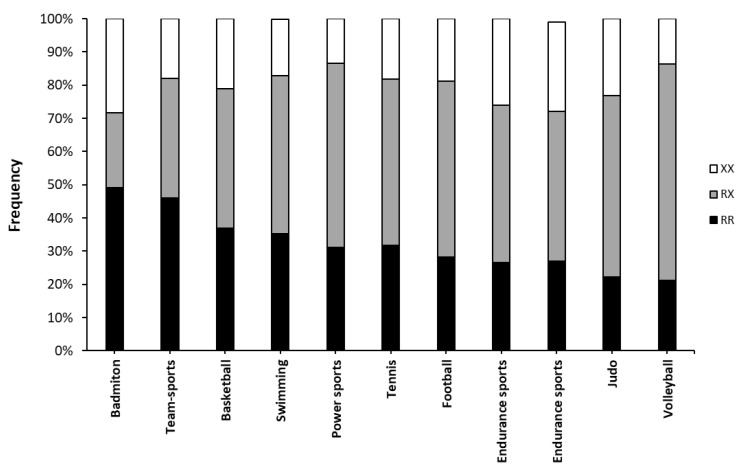
Frequencies of *ACTN3* genotypes in samples of European professional athletes. Data have been obtained from Eynon et al. [23] for team sports, from Garatachea et al. [21] for basketball, from Ruiz et al. [16] for swimming, from Eynon et al. [32] for power sports, from Moreno-Perez et al. [20] for tennis, from Del Coso et al. [38] for football, from Eynon et al. [32] and Lucia et al. [14] for endurance sports, from Rodriguez-Romo et al. [39] for judo and from Ruiz et al. [40] for volleyball.

This is the first study showing the distribution of the *ACTN3* R577X genotypes in a sample of professional badminton players. The distribution found in this study with a surprisingly predominance of the RR genotype (~50%) is different from the distribution found by other authors in non-athletic populations of European participants where the predominance corresponded to the RX genotype (41–51% of non-athletic controls are RX) [23,32]. Additionally, the frequency of these European RR badminton players was higher than the one found in the European population of the 1000 genome database, where RR represents only 31% of Europeans [31]. Curiously, the frequency of RR individuals in the current sample of badminton players is the highest when compared to other samples of European athletes of different sports modalities (Figure 2). For instance, the frequency of RR badminton players was higher than the one found in elite Spanish team sport athletes [23] and higher than in other sports with a similar intermittent nature such as tennis [20], basketball [21] and volleyball [16]. Of all the sports included in Figure 2, the one with a higher association with badminton is tennis, due to the similarities in the duration of the rallies, the use of a racket to hit a moving object and the patterns of movements performance. Moreno-Perez et al. [20] found a predominance of the RX genotype in a sample of elite tennis players and concluded that it was probable that the *ACTN3* genotype was not associated with elite tennis performance. These authors argued that tennis is a sport in which performance is more associated with technical components and skills (respect to other “purer” power modalities such as athletics or weightlifting), which are less influenced by genetics. The data provided by this investigation are innovative because, in badminton, a sport with high relevance of technical drills and skills, the possession of the RR genotype may be a factor predisposing to one achieve the status of the elite athlete. These data could indicate that badminton is a sport where obtaining high values of explosive force is transcendental to achieve greater sports performance, as most of the actions leading to the obtaining of a point are associated with the production of explosive force. Yang et al. [5] suggested that the effect of *ACTN3* genotype on sports performance differs between male and female athletes, indicating that the relative effect of α-actinin-3 deficiency on muscle power is reduced in males (as they found that no sprint athlete competing at the Olympic level was XX). However, there is a discrepancy regarding these findings since other authors have not found differences in the distribution of the *ACTN3* genotypes between men and women athletes of the same level [33]. In the case of badminton, the distribution was similar between men and women with a predominance of the RR genotype in both sexes, suggesting that for badminton, the benefits of possessing the RR genotype seem to be similar for both men and women.

Several authors who have compared the distribution of *ACTN3* genotypes in elite athletes of different sports modalities have found that, in power-oriented sports, the percentage of elite athletes with an XX genotype is lower than in endurance sports and lower than in non-athletic populations [5,34]. It should be noted that none of the badminton players who have reached a top-ten position in the World Badminton Federation ranking possessed the *ACTN3* XX genotype. From a practical standpoint, the complete deficiency of α-actinin-3 due to the XX genotype could be a restrictive factor to achieving maximum performance in badminton, at least at the worldwide level. Badminton is a sport with very explosive movements and eccentric muscular contractions that cause high values of exercise-induced muscle damage [26]. Several studies have found that individuals with the XX genotype are more prone to exercise-induced muscle damage (including higher levels of force loss during exercise and increased muscle pain [35,36]), and they have a less adaptative response to training [37]. The lack of XX badminton players within the players ranked in the world’s top-ten ranking could be linked to the lower capacity of XX athletes to resist muscle strain, as a phenotype associated with the lack of α-actinin-3 in fast-type muscle fibers.

Sports performance in badminton depends on many factors such as technique, tactics and physical and psychological aspects [24,27,41]. Genetics is also among these performance factors, as it may contribute to the innate possession of phenotypes that predispose one to certain physical and physiological conditions. Thus, although the *ACTN3* RR genotype seems to favor “pure” power and speed sports [5,6], its importance in skill-based sports (e.g., racquet sports, team sports) may be diluted as other facets also condition performance [20,23]. In contrast, in cyclical sports where tactical aspects and strategy may have a limited influence [5], the possession of a genotype that predisposes one to produce powerful contractions may be a key factor for performance. In any case, knowing the genotype of an athlete can have many benefits independently of the type of sport. The knowledge of the players’ *ACTN3* genotype may benefit coaches as they can adapt their training routines to the player’s genotype, being able to optimize training adaptations or to prevent injuries. For instance, a badminton player with the *ACTN3* XX genotype could need more explosive strength training to alleviate the deficiencies generated by the complete deficiency of the α-actinin-3 [42,43]. Hence, in the opinion of these authors, an XX badminton player may reach world’s top-ten ranking with optimized training structures associated with his/her genotype/phenotypes.

There are some limitations to this study that should be explained to understand the reach of the study outcomes. First, the sample size was small for a genetic relationship study, especially when establishing the different categories of professional badminton players and for the sub-sample of women. However, the nature of the sample (European elite badminton players) is the main reason for the size of the sample, as only a few dozen players can be categorized as professional and with the status of elite badminton player in Europe. Although we were able to recruit more than half of the players competing in the 2018 European Championship, the findings of this study should be confirmed in larger samples of badminton players, such as the ones classified for a World Championship. Second, this study does not have a control group of European participants, but we used data from the 1000 genome database to find ethnically matched controls [31]. This comparison with European non-athletic controls has been key in demonstrating the overrepresentation of the RR genotype in European elite badminton players. Nevertheless, a comparison with samples of amateur or lower-level badminton players may help to clarify if the RR genotype is beneficial for reaching the status of elite athlete. Third, the study was conducted with European badminton players, so the results cannot be extrapolated to badminton players from other continents where the distribution of the *ACTN3* gene genotypes may be different due to ethnicity. Interestingly, it has been recently found that the influence of the *ACTN3* R allele may be higher for athletes of Western countries and women, while the RX genotype may be the variant more beneficial for sports performance in Asian athletes of power sports [7]. Additionally, Yang et al. [44] found no evidence of an association between α-actinini-3 deficiency and sprint/power performance in African athletes due to the low frequency of the XX genotype in both non-athletic controls and athletes. Last, other candidate genes and epigenetic modifications may also contribute to the attainment of elite status in badminton, as several physiological, anthropometrical and psychological traits contribute to elite performance [45]. Hence, the possession of a certain *ACTN3* genotype should not be interpreted as the only heritable factor for badminton performance, as other genes and epigenetic modifications may predispose players to have a natural talent for badminton and/or to have an enhanced response to physical training. Collectively, we must be cautious when establishing conclusions regarding the definitive relationship between the *ACTN3* gene and performance in badminton players, since genetic studies with small samples may contain hidden biases, such as population stratification, which may increase the risk of making a type II error.

## 5. Conclusions

In conclusion, the *ACTN3* RR genotype was more frequent in professional badminton players competing in a European Championship than in samples of ethnically matched controls. The overrepresentation of the *ACTN3* RR genotype was similarly present in male and female professional badminton players. Interestingly, none of the badminton players ranked in the top ten of the world ranking possessed the *ACTN3* XX. These outcomes suggest that the expression of functional α-actinin-3, only present in players with the RR and RX genotypes, may confer an advantage for reaching the status of elite athlete in badminton, and especially the world’s top-ten ranking. Still, large-scale studies with different ethnic backgrounds are needed to confirm the association of the R allele of *ACTN3* with badminton performance.

## Figures and Tables

**Table 1:** Number (frequency, in %) of professional badminton players according to their *ACTN3* genotypes (RR, RX and XX) and their allelic distribution (R and X), in comparison to the 1000 genome database of ethnically matched controls [31] (only frequency, in %).

Group	Genotype Frequency			Allele Frequency	
	RR	RX	XX	R	X
All n = 53	26 (49.1)	12 (22.6)	15 (28.3)	64 (60.4)	42 (39.6)
Men n = 31	14 (45.2)	8 (25.8)	9 (29.0)	36 (58.1)	26 (41.9)
Women n = 22	12 (54.5)	4 (18.2)	6 (27.3)	28 (63.6)	16 (36.4)
1000 genome database; European	31.0	51.1	17.9	56.6	43.4

**Table 2:** Association between the *ACTN3* genotypes and categories in professional European badminton players.

	Top 20		Top 50		Top 100		All	
	*p*-Value	Cramer’s V	*p*-Value	Cramer’s V	*p*-Value	Cramer’s V	*p*-Value	Cramer’s V
Top 10	0.615	0.348	0.523	0.289	0.183	0.283	0.144	0.261
Top 20	–	–	1.000	0.000	1.000	0.076	0.905	0.080
Top 50	–	–	–	–	0.876	0.089	0.698	0.098
Top 100	–	–	–	–	–	–	0.967	0.028

## Data Availability

The data presented in this study are available on request from the corresponding author. The data are not publicly available due to legal restrictions.
